# Finite Element Assessment of the Screw and Cement Technique in Total Knee Arthroplasty

**DOI:** 10.1155/2020/3718705

**Published:** 2020-10-15

**Authors:** Chong Zheng, Hai-yang Ma, Yin-qiao Du, Jing-yang Sun, Ji-wei Luo, Dong-bin Qu, Yong-gang Zhou

**Affiliations:** ^1^Department of Orthopaedics, Nanfang Hospital, Southern Medical University, Guangzhou, China 510515; ^2^Department of orthopaedics, Chinese People's Liberation Army General Hospital, 28 Fuxin Road, Haidian District, Beijing, China 100853

## Abstract

**Background:**

The screw and cement technique is a convenient method used to rebuild medial tibial plateau defects in primary total knee arthroplasty (TKA). The objective of this study was to perform a finite element assessment to determine the effect of different numbers of screws on the stability of TKA and to determine whether differences exist between two different insertion angles.

**Method:**

Six tibial finite element models with defects filled with screws and cement and one model with defects filled only with cement were generated. Contact stresses on the surface of cancellous bone in different areas were calculated.

**Results:**

Compared to the cement-only technique, the stress on the border of cancellous bone and bone cement decreased by 10% using the screw and cement technique. For bone defects with a 12% defect area and a 12-mm defect depth, the use of 1 screw achieved the greatest stability; for those with a 15% defect area and a 20-mm defect depth, 2 screws achieved the greatest stability.

**Conclusions:**

(1) The screw and cement technique is superior to the bone cement-only technique. For tibial defects in which the defect area comprises a large percentage but the depth is less than 5 mm, the screw and cement technique is recommended. (2) Vertical screws can achieve better stability than oblique screws. (3) Screws should be used in moderation for different defects; more is not always better.

## 1. Introduction

Medial tibial plateau defects can often be found in primary total knee arthroplasty (TKA) and require additional management to ensure implant stabilization, support, and durability. Many techniques have been used, including cement, metal augmentation [1–5], bone grafts (autografts or allografts) [1, 2, 6–10], and the screw and cement technique [11–13]. Compared to other techniques, the screw and cement technique has many advantages, namely, it is less expensive, easier to perform, and less time-consuming. Ritter [11–13] reported successful results at early, intermediate, and long-term follow-up after use of the screw and cement technique to correct large tibial defects (5-30 mm). However, in previous studies, the number and insertion angle of screws were chosen based on personal experience, to fill a given medial tibial defect, previous authors have used as many screws as possible to ensure the stability of the tibial prosthesis [14–18], and no published study has determined the optimal number of screws or whether differences exist between the two different frequently used screw insertion angles. The purpose of this study was to perform a finite element (FE) assessment to determine the effect of different numbers of screws on the stability of TKA for two types of moderate uncontained type-2 defects and to determine whether differences exist between two different screw insertion angles.

## 2. Materials and Methods

A knee of a healthy volunteer (height 1.73 m, weight 60 kg, male) was scanned by computed tomography (CT), and a geometric knee model was built using the Mimics 11 software. The composition of each model is shown in Table 1. The performance of the component materials in each model is shown in Table 2. Then, based on the geometric knee model and the area percentage and depth statistics of tibial defects summarized for patients who underwent TKA using screws and cement due to medial plateau defects, we simulated two types of familiar defects with a 12% defect area (12-mm depth) and a 20% defect area (15-mm depth) after performing a horizontal resection of 11 mm (according to clinical experience) above the tibial plateau. The defect depths were two common depths that were measured during the operations of 40 patients whose tibial defects were treated using screws and cement.

**Figure 1:**
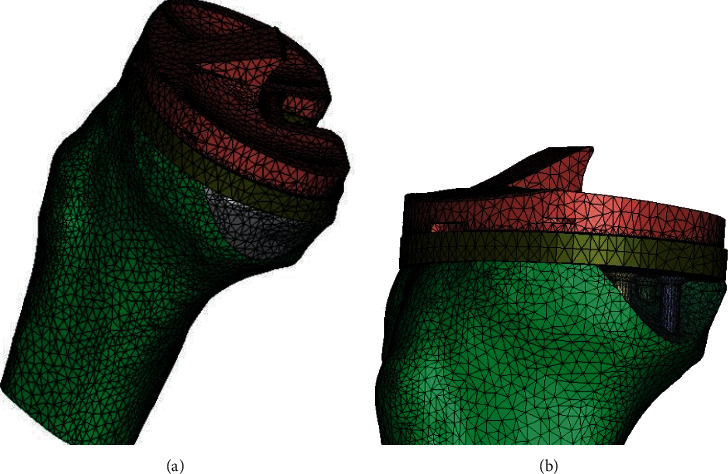
(a) TKA meshed block model with a 20% defect area built with three screws and cement. (b) TKA meshed block model with a 20% defect area built with three screws and cement (cement removed).

Based on the two tibial defect models above, 7 three-dimensional, static, proximal tibial FE models implanted with a tibial prosthesis (PFC sigma@Dypu) and a plastic insert were built using the Mimics 11 software (Figures 1(a) and 1(b)). The selected prosthesis is one frequently used by our senior surgeon. The diameter of the screw was 6.5 mm; the distance between the upper surface of the screw head and the lower surface of tibial component was 0 mm.

The modulus of the cortical bone was used by Frehill et al. [14], and it is within the modulus range cited and used by other authors. The value of the cancellous modulus used is within the range of values (389-1132 MPa) cited and obtained experimentally by Au et al. for cancellous bone [15].

The contact between the bearing and tibial tray was modelled using a surface-to-surface contact algorithm, and a constant coefficient of friction (0.1) was used in all models [16].

**Figure 2:**
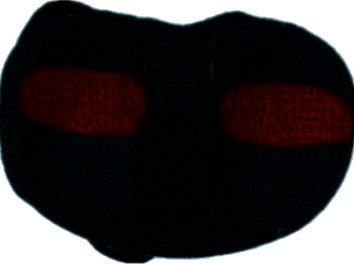
Pressure load application areas.

The load application area is shown in Figure 2. Traditionally, the loads applied to the knee to represent a level gait in FE modelling have been 2.5-3 times the body weight [15, 18, 19]. These data are based on models of knee biomechanics developed by Morrison and Morrison [17]. Recently, somewhat lower levels of loading (2.2 times the body weight) have been measured in vivo [20], and it seemed more appropriate to use these loads in the present study. Thus, a total load of 1294 N (representing a 60-kg person) was used in this study. In all models, the distal end of the tibia was assumed to be constrained in all directions.

**Figure 3:**
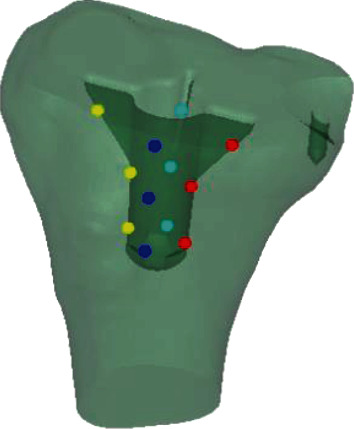
Dark blue: three anterior spots (A). Light blue: three posterior spots (P). Yellow: three lateral spots (L). Red: three medial spots (M).

**Figure 4:**
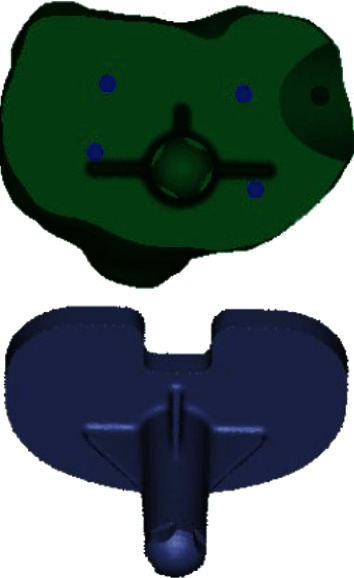
Trisection point of the midcourt line of the medial and lateral tibial plateau.

Contact stresses on the surface of cancellous bone were measured using Abaqus 6.12 software to determine whether the use of screw(s) and cement to fill proximal medial defects would result in an increased likelihood of bone failure due to increased stresses. The critical level for this stress was considered to be 2.8 MPa (equivalent to approximately 4000 *με*) [21]. This very conservative value is one of the lowest values in the literature. Cancellous bone stresses were also examined to ensure that reduced stresses did not lead to severe bone resorption using an adopted resorption threshold of 0.1 MPa (equivalent to approximately 150 *με*) [21]. To assess whether differences exist among the stresses on the surface of cancellous bone under different repair schemes, stresses at 12 locations (3 points at each of the medial, lateral, anterior, and posterior locations) were measured (Figure 3) on the surface of cancellous bone in the medullary cavity. Additionally, stresses at the 4 trisection points of the midcourt line of the medial and lateral plateau (Figure 4), stress focus locations around the screws, and stresses on the surface of the defects were measured (6 points at each of the medial, lateral, anterior, and posterior locations).

## 3. Results

**Figure 5:**
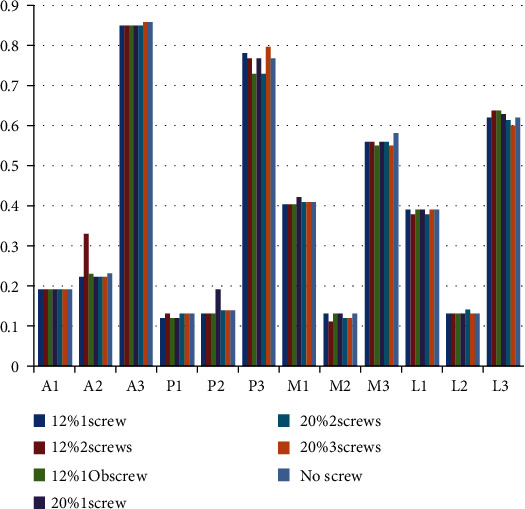
Stresses (MPa) at 12 spots on the surface of cancellous bone in the medullary cavity.

The stresses at 12 points on the surface of cancellous bone in the medullary cavity of each model are shown in Figure 5. No significant difference was found, and all stresses measured were within the normal range (0.1-2.8 MPa).

**Figure 6:**
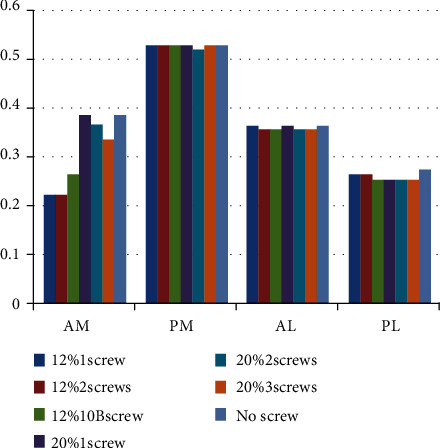
Stresses (MPa) at 4 trisection points on the medial and lateral plateau. AM: anteriomedial; PM: posteromedial; AL: anterolateral; PL: posteromedial; OB: oblique.

The stresses at 4 the trisection points are shown in Figure 6. The stresses of the anteromedial trisection points in models with a 12% defect area (0.22-0.26 MPa) were lower than those in models with a 20% defect area (0.33-0.38 MPa). However, no other statistically significant difference was found.

Table 3 shows the stresses at the focus points that exist in the cancellous bone around the screws. All stresses were within the safety range (0.1-2.8 MPa). In models with a 12% defect area and a 12-mm depth, the use of 1 vertical screw to rebuild the defect resulted in a lower focused stress (1.05 MPa) than the stress found with the use of 1 oblique screw (1.23 MPa). In models with a 20% defect area and a 15-mm depth, the use of 3 screws resulted in a higher focused stress (1.77 MPa) than that resulting from the use of 1 or 2 screws (1.66 MPa and 1.71 MPa, respectively).

**Figure 7:**
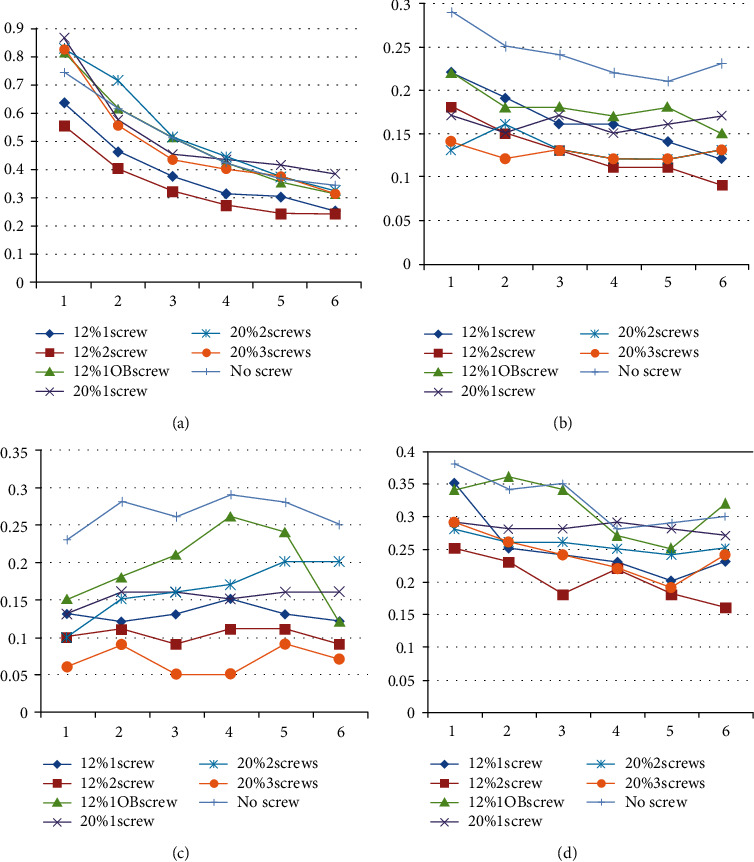
Stresses on the surface of defects (MPa). (a) Six anterior spots on the surface of defects. (b) Six posterior spots on the surface of defects. (c) Six medial spots on the surface of defects. (d) Six lateral spots on the surface of defects.

As shown in Figure 7, each defect was divided into 4 sections (medial, lateral, anterior, and posterior), and the stresses at 6 points were measured in each section. The results show that, compared to the cement-only technique, the use of 1 vertical screw combined with bone cement to repair the defect (area of 12%, depth of 12 mm) resulted in a 32% reduction in the stress on the surface of the defect (anterior 22%, posterior 22%, medial 52%, lateral 21%), while the use of 1 oblique screw combined with bone cement to repair the defect (area of 12%, depth of 12 mm) resulted in a 15% reduction of the stress on the surface of the defect (anterior 0%, posterior 0%, medial 30%, lateral 3%). Compared to the use of one oblique screw combined with bone cement to repair the defect (area of 12%, depth of 12 mm), the use of 1 vertical screw reduced the stresses on the surface of the defect by 20% (anterior 26%, posterior 26%, medial 31%, lateral 19%). In models with a 12% defect area and a 12-mm depth, the use of 2 screws compared to 1 vertical screw resulted in lower stresses on the surface of the defects; however, the stresses on the medial side were less than 0.1 MPa, which can lead to severe bone resorption. Finally, when comparing models with a 20% defect area and a 15-mm depth with models with a 12% defect area and a 12-mm depth, a greater defect range resulted in greater stress on the surface of the defect when using the same number of screws.

## 4. Discussion

Medial tibial plateau defects are common in complex primary TKA, and for defects less than 10 mm, resection of the tibial plateau allows for complete removal of the defect without requiring further procedures [22]. However, in deeper and larger lesions, tibial resection of more than 12 mm may damage ligamentous structures. It has been observed that increasing the stress on the proximal tibia [4, 23] will cause many other problems, such as the need for a thicker tibial insert and patellar joint complications [23]. Berend found that using a thicker tibial insert would not cause direct surgical failure, but increased tibial resection and ligament imbalance may result in an increased failure rate [24]. Thus, for defects with a depth of more than 10 mm, other reconstruction methods need to be used. In this study, after making a horizontal resection of 11 mm, the defect depths were 12 and 15 mm; the author chosen these two defects based on data of 40 patients measured during TKA and got good clinical outcome after up to 10 years follow-up.

There are 5 types of basic reconstruction methods, including tibial component downsizing and resection of uncapped proximal medial bone, the cement-only technique, the screw and cement technique, metal augmentation, and autologous bone grafting [5–7, 13, 25, 26]. Compared to other methods, the screw and bone cement technique has many advantages as follows: (1) compared to the bone cement-only method, the strength of bone cement is greatly enhanced; (2) compared to bone grafting and metal augmentation, the screw and cement technique can simplify the operation, shorten the operative time, decrease the risk of infection, and reduce the use of additional implants; and (3) it is less expensive, and the effect is reliable. Although Brooks' in vitro biomechanical experiments reported that the use of the screw and bone cement technique in repairing defects of greater than 5 mm was associated with potential problems [27], Ritter first applied the screw and cement technique in clinical practice and obtained satisfactory short-term results [11]. This team further proceeded with medium- and long-term follow-up and obtained satisfactory results [12, 13].

In Brooks' [27] in vitro biomechanical experiments, tibial defects were rebuilt using bone cement only, bone cement combined with 2 screws, a stainless steel wedge, a Plexiglas wedge, and an integral metal custom-made component. The best results were found for the integral metal custom-made component, followed by the results for the metal wedge and Plexiglas wedge, and the worst results were observed for bone cement only. Bone cement combined with 2 screws showed only little improvement compared to the results for bone cement only. However, no FE analysis has been performed to demonstrate these results. In this study, based on clinical experience, we generated two types of defects, which are often observed clinically, in the FE model of the tibial plateau. They then used different strategies to repair the defect, analysed the questions regarding screw number and insertion direction, and obtained valuable conclusions.

The load application area (Figure 2) used was based on the conditions that occur in the late stance phase of gait, where maximum joint reaction occurs [17], and was determined from the work of Villa, who evaluated contact locations using Fuji Prescale pressure-sensitive films and in vitro TKA models [16]. This phase of the gait produces the highest stresses in the proximal bone and ligament. Loading was applied as a uniform pressure load to selected surfaces of the bearing where the medial and lateral femur condyles would make contact. The gastrocnemius muscle is the only active muscle in this late stance gait phase. As the gastrocnemius muscle does not attach to any region of the proximal tibia, it was not necessary to include any ligaments or muscles in the models. The effect of the gastrocnemius, however, is represented by the applied joint reaction force.

In this FE study, the load on the plateau was 2.2 times the body weight, and the patient's body weight was 60 kg. Based on the mechanical result of each model, we found that the use of more screws can achieve lower stresses on the surface of the defect. However, more screws may cause stress shelter. In the model consisting of a 12% defect area and a 12-mm defect depth, the use of 2 or more screws caused stress shelter, and in the model consisting of a 20% defect area and a 15-mm defect depth, the use of 3 or more screws caused stress shelter. However, in patients with a higher body weight, the load on the plateau will increase, and use of the same number of screws may not cause stress shelter. To test this conjecture, we increased the load to 4000 N. The results showed that the stress on the same points, which was below the safety range of <1294 N, increased to the safety range. Furthermore, the body weight corresponding to 4000 N on the plateau was approximately 185 kg, and this weight is rare in Chinese patients. Therefore, it can be concluded that, in patients over 60 kg, the optimal number of screws will increase, and the increased quantity will not exceed 1. In clinical practice, different patients have different body weights, tibial plateau sizes, and defect characteristics. This study does not include all situations, and patient-specific FE models could be built to further elucidate the optimal screw number.

In this study, we found that the vertical screw direction was superior to the oblique screw direction in terms of mechanical stability; therefore, the vertical screw direction is recommended in clinical practice.

In this study, the diameter of screws was 6.5 mm. And the upper surface of the screw head was on the same level of tibial platform which touched the lower surface of tibial component. These were the same with our senior surgeon's clinical practice, and we acquired good long-term clinical outcome. We believed there would be effect of different diameters, material, and distance of screw from the implant on the stresses, while our finite models were limited, we would study this question in the future. We believe our study may provide a surgical guidance to surgeons while performing TKA for patients with tibial bone defects.

This study did not consider all of the prosthesis types and every screw angle, but it can be a good reference in clinical practice. Further study will be conducted in the future.

## Figures and Tables

**Table 1:** The composition of each model.

	Model 1	Model 2	Model 3	Model 4	Model 5	Model 6	Model 7
Number of screws	1	2	1	1	2	3	0
Defect area%	12%	12%	12%	20%	20%	20%	12%
Depth (mm)	12	12	12	15	15	15	12

Screw of model 3 was placed in oblique direction, and screws of the other models were placed in vertical direction.

**Table 2:** The performance of component materials in each model.

Material	E (GPa)	v
PMMA (cement)	2.27	0.46
Ti6Al4V(prosthesis)	110	0.3
Cortical bone	17	0.3
Cancellous bone	0.7	0.3
UHMWPE (tibial insert)	2.3	0.25
Titanium alloy(screw)	110	0.3

**Table 3:** Stress focus spots around the screws (MPa).

	Screw 1	Screw 2	Screw 3
12% 1 screw	1.05		
12% 2 screws	0.70	1.60	
12% 1OB screw	1.23		
20% 1 screw	1.66		
20% 2 screws	0.91	1.71	
20% 3 screws	1.28	1.77	1.68
No screw	—	—	—

## Data Availability

The data used to support the findings of this study are included within the article.
